# Phytohormones Related to Host Plant Manipulation by a Gall-Inducing Leafhopper

**DOI:** 10.1371/journal.pone.0062350

**Published:** 2013-04-30

**Authors:** Makoto Tokuda, Yusuke Jikumaru, Keiichiro Matsukura, Yumiko Takebayashi, Shun Kumashiro, Masaya Matsumura, Yuji Kamiya

**Affiliations:** 1 Laboratory of System Ecology, Faculty of Agriculture, Saga University, Saga, Japan; 2 Growth Regulation Research Group, RIKEN Plant Science Center, Yokohama, Japan; 3 Agro-Environment Research Division, Kyushu Okinawa Agricultural Research Center, National Agriculture and Food Research Organization; Koshi, Kumamoto, Japan; 4 The United Graduate School of Agricultural Sciences, Kagoshima University, Kagoshima, Japan; United States Department of Agriculture, Agriculture Research Service, United States of America

## Abstract

The maize orange leafhopper *Cicadulina bipunctata* (Hemiptera: Cicadellidae) induces galls characterized by growth stunting and severe swelling of leaf veins on various plants of Poaceae. Previous studies revealed that galls are induced not on feeding site but on distant, newly extended leaves during the feeding, and strongly suggested that some chemicals injected by the leafhopper affect at the leaf primordia. To approach the mechanism underlying gall induction by *C. bipunctata*, we examined physiological response of plants to feeding by the leafhopper. We performed high-throughput and comprehensive plant hormone analyses using LC-ESI-MS/MS. Galled maize leaves contained higher contents of abscisic acid (ABA) and *trans*-Zeatin (tZ) and lower contents of gibberellins (GA_1_ and GA_4_) than ungalled maize leaves. Leafhopper treatment significantly increased ABA and tZ contents and decreased GA_1_ and GA_4_ contents in extending leaves. After the removal of leafhoppers, contents of tZ and gibberellins in extending leaves soon became similar to the control values. ABA content was gradually decreased after the removal of leafhoppers. Such hormonal changes were not observed in leafhopper treatment on leaves of resistant maize variety. Water contents of galled leaves were significantly lower than control leaves, suggesting water stress of galled leaves and possible reason of the increase in ABA content. These results imply that ABA, tZ, and gibberellins are related to gall induction by the leafhopper on susceptible variety of maize.

## Introduction

Many herbivores have an ability to manipulate their host plant morphologically and physiologically for their own benefit [1]. Gall induction by various insects is a typical example of such manipulations [2]–[3]. Many researchers have been interested in the mechanism underlying the host plant manipulation by gall-inducing insects. Although previous studies have been succeeded to induce morphological changes in plant tissues by the artificial application of extract from gall-inducing insects [4]–[7], fundamental mechanism underlying the gall induction by insects is still unknown [8]. This is due to the difficulty in constructing laboratory bioassay systems for most gall-inducing insects [9]–[14].

Some previous studies strongly suggested the involvement of chemical stimuli secreted from insects, because the site of gall induction is different from the feeding sites of gall-inducing insects in some plant-gall inducer systems [12], [15]. In addition, a previous study reported plant regulators stimulating cell division and inducing neoplasm formation on pods of *Pisum sativum* L. (Fabaceae), which is derived from pea weevil *Bruchus pisorum* L. and *Callosobruchus maculates* F. (Coleoptera: Bruchidae) [16]. However, unlike gall induction by insects, the neoplasm induction by the pea weevil has negative effect to the inducer [16], and the event may not be generalized for gall induction by insects [17].

The maize orange leafhopper *Cicadulina bipunctata* (Melichar) (Hemiptera: Cicadellidae) induces growth stunting and leaf galls characterized by the severe vein swelling on various Poaceae [18]–[23]. Though previous studies attributed the symptoms to a leafhopper-transmitted virus [24]–[28], recent studies strongly suggest that some chemicals injected by *C. bipunctata* during feeding are responsible for the gall induction [12]–[13], [18], [29]–[30]. Similar to other gall-inducing insects, the gall induction of *C. bipunctata* is adaptive for the inducer because free amino acids and glucose contents increased significantly in galled leaves and it results in the faster development and higher survival rate of offspring growing on the plant [31].

The leafhopper usually feeds on mature host leaves and galls are induced not on feeding sites but on distant, newly developing leaves at approximately one week after the initiation of feeding [12]–[13], [23], [31]. Because the degrees of growth stunting and leaf-vein swelling were significantly correlated with infestation density and length, the leafhopper is considered to induce the symptom by a dose-dependent reaction [12]–[13]. Galls are induced more severe when plants were attacked at younger stages [32]. Both nymphs and adults of *C. bipunctata* have the ability to induce galls on host plants [13]. A recent examination using barley chromosome disomic addition lines of wheat revealed that the degrees of growth stunting and leaf-vein swelling were not significantly correlated, implying that the two symptoms are independent phenomena even though both are initiated by the feeding of *C. bipunctata* [23].

This leafhopper is an ideal study material to clarify the mechanism of gall induction by insects because of the following reasons: Mass-rearing techniques have already been established for this leafhopper [33]; feeding site and gall-inducing site are different in this leafhopper, which enables us to separate the chemical examination at the site of feeding and gall induction [12]; model plants such as rice *Oryza sativa* L. and wheat *Triticum aestivum* L. are readily available as hosts in various experiments [11], [23]; and comparative studies using non-galling leafhoppers as well as resistant plant varieties can be performed [31].

In this paper, we focus on the physiological response of plants at the site of gall induction to elucidate the mechanism of morphological manipulation of plant leaves by the leafhopper. Using both susceptible and resistant varieties of maize to gall induction by the leafhopper, phytohormone dynamics in leaves that will exhibit the symptom of gall are comprehensively analyzed and water contents of leaves are also examined.

## Materials and Methods

### Insect Stock Culture and Maize Varieties

A stock culture of *C. bipunctata* originally collected from Kyokushi, Kumamoto, Japan (32.57° N, 130.50°E) in September 2000 was used for experiments. The leafhopper was reared on rice seedlings at 25°C under an LD 16∶8 h photocycle using the method described by a previous study [33].

Two maize (*Zea mays* L.) varieties ‘3081’ and ‘30D44’ were used in the experiments. The former is a variety susceptible to feeding by *C. bipunctata* [12], [31] and the latter has a high resistance to feeding by the leafhopper [31]–[32]. The growth stunting and gall-inducing profile by *C. bipunctata* on the susceptible variety 3081 was examined in previous studies [12]–[13], [31]: when the seedlings at the second leaf stage were infested by five males of *C. bipunctata* for eight days, the plant growth was significantly stunted and veins of the third leaf were severely swollen. However, the resistant variety 30D44 displays only weak symptoms of growth stunting and leaf vein swelling, even if seedlings were fed on by *C. bipunctata* [31]–[32].

### Feeding Experiments

Using the method described in a previous study [12], maize seeds were individually sowed in plastic cups (220 mL) with soil and kept in the phytotron at 25°C under an LD 16∶ 8 h photocycle. All maize seedlings were covered by acrylic cylinders (4.5 cm diameter and 24.5 cm deep) with a nylon cloth for ventilation on the top.

Seven days after sowing, seedlings at the second leaf stage were randomly separated into the following four categories: (1) five adult males of *C. bipunctata* were released on a seedling from the 7th to 15th days ( = leafhopper treatment for eight days); (2) no adult males of *C. bipunctata* were released ( = control); (3) five adult males of *C. bipunctata* were released on a seedling from the 7th to 11th days (early treatment for four days); and (4) five adult males of *C. bipunctata* were released on a seedling from the 11th to 15th days (late treatment for four days). In the experiment, we did not use females of *C. bipunctata* to avoid possible influences of their ovipositions into seedlings. Adults can be sexed easily based on the presence or absence of a black ovipositor on the ventral surface of the abdomen. In the categories (1) and (2), plants were dissected 48, 96, 144 or 192 hours after the release of leafhopper adults and the third leaf, which is expected to exhibit galls [12], were weighted and immediately freezed in liquid nitrogen for comprehensive phytohormone analysis. Similarly in the categories (3) and (4), the third leaf samples were prepared 144 or 192 hours after the release of leafhoppers. Six replications were conducted for respective categories and times.

### Quantification of Phytohormones

Extraction and purification of IAA, isopentenyladenine (iP), *trans*-zeatin (tZ), GA_1_, GA_4_, ABA, jasmonic acid (JA), jasmonoyl isoleucine (JA-Ile) and salicylic acid (SA) were performed by solid-phase extraction. Stable isotope-labeled compounds used as internal standards were: d2-IAA (Sigma-Aldrich); d6-iP, d5-tZ, d2-GA_1_, d2-GA_4_ (Olchemim Ltd, Olomouc, Czech Republic); D6- ABA (Icon Isotopes, Summit, NJ, USA); d6-SA (Sigma-Aldrich); and d2-JA (Tokyo Kasei, Tokyo, Japan). 13C6-JA-Ile was synthesized as described in a previous study [34].

For simultaneous measurement of phytohormones, approximately 100 mg fresh weight of the third maize leaf was lyophilized, ground with 10-mm zirconia beads, and extracted 2 times with a total of 10 volumes of 80% (v/v) methanol containing 1% (v/v) acetic acid with internal standards at 4°C overnight. Extracts were centrifuged at 4°C, 14,000* g*, 10 min, and the supernatant was collected. The supernatant was evaporated to water containing 1% acetic acid, and applied to a pre-equilibrated Oasis HLB column cartridge (30 mg, 1 ml, Waters, Milford, MA, USA). After washing with 1 ml of water containing 1% (v : v) acetic acid, hormones were eluted with 2 ml of 80% (v : v) acetonitrile containing 1% (v : v) acetic acid, and then acetonitrile were evaporated in vacuo (e. g., less than 400 µl at 2 ml of 80% acetonitrile containing 1% AcOH) to give extract in acidic water. Extract were applied to a pre-equilibrated Oasis MCX column cartridge (30 mg, 1 ml, Waters). After washing the cartridges with 1 ml of water containing 1% acetic acid, the acidic and neutral fraction containing IAA, GA_1_, GA_4_, ABA, JA and JA-Ile was eluted with 2 ml of 80% acetonitrile. Two hundred microliters of this fraction was transferred, dried, and reconstituted with water containing 1% acetic acid for analysis of SA. The MCX cartridges were further washed with 1 ml of 5% (v : v) aqueous ammonia, and the basic fraction containing iP and tZ was eluted with 2 ml of 60% (v : v) acetonitrile containing 5% (v : v) aqueous ammonia. After removing acetonitrile in vacuo, acidic and neutral fractions were further applied to a pre-equilibrated Oasis WAX column cartridge (30 mg, 1 ml, Waters). After washing with 1 ml of 1% acetic acid and 2 ml of 80% acetonitrile, the acidic fraction containing IAA, GA_1_, GA_4_, ABA, JA and JA-Ile was eluted with 2 ml of 80% acetonitrile containing 1% (v : v) acetic acid. This fraction was dried and reconstituted with 1% acetic acid. Hormones were analysed by LC-electrospray ionization (ESI)-MS / MS (Agilent 6410) on a ZORBAX Eclipse XDB-C18 column (Agilent) as described in a previous study [35], and quantified using MassHunter v. B. 01. 02 spectrometer software (Agilent, Santa Clara, CA, USA).

### Leaf Water Content

Feeding experiments of the abovementioned categories (1) and (4) were performed for examining water contents in leaves. Plants were dissected 192 hours after the release of leafhoppers and the third leaf was sampled. The leaves were dried for 24 hours at 50°C after the measurement of fresh weight. The water contents (% fresh weight) of the third leaf were calculated from the fresh and dry weights of each leaf. Ten replications were conducted for each category.

### Statistical Analyses

The concentration of each phytohormone was examined in each variety by analysis of variance (ANOVA) between treatments and control at 48, 96, 144, and 192 hours after the release of leafhoppers). Leaf water contents were analyzed in each variety by ANOVA after all percentage data were arcsine-transformed. In multiple comparisons, treatment means were compared by Tukey’s honestly significant difference (HSD) test.

## Results

**Figure 1:**
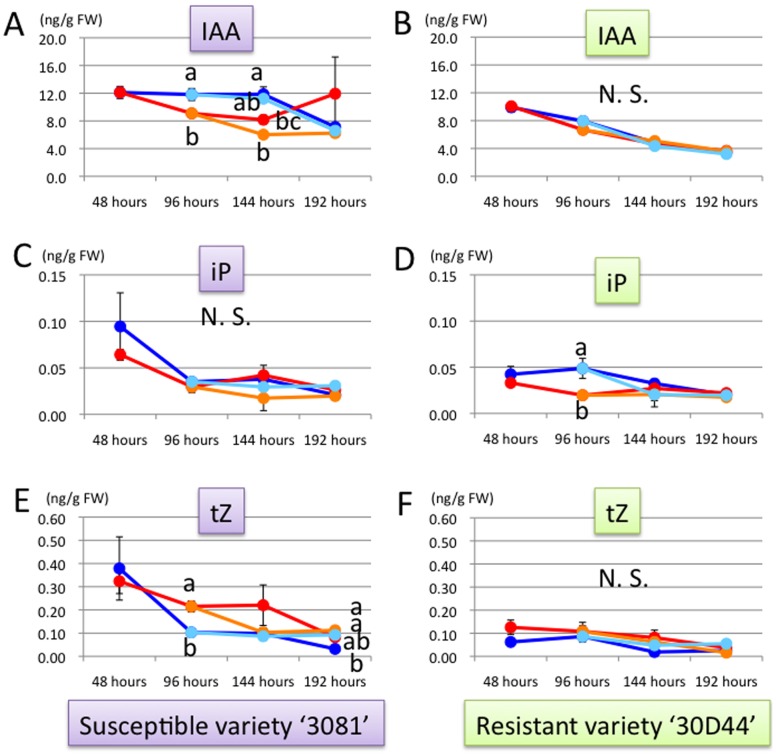
Changes in IAA, iP and tZ concentrations in the third leaf of maize. (A) IAA concentration (ng/FW) in the variety ‘3081’. (B) IAA concentration (ng/FW) in the variety ‘30D44’. (C) iP concentration (ng/FW) in the variety ‘3081’. (D) iP concentration (ng/FW) in the variety ‘30D44’. (E) tZ concentration (ng/FW) in the variety ‘3081’. (F) tZ concentration (ng/FW) in the variety ‘30D44’. Line colors indicate leafhopper treatment for eight days (red lines), control (blue lines), early leafhopper treatment for four days (orange lines), and late leafhopper treatment for four days (light blue lines). Different letters indicate significant differences between control and treatments at each time point (ANOVA; followed by Tukey’s HSD test at 144 and 192 hours).

In the susceptible variety 3081, IAA concentration did not clearly change by the leafhopper treatment. Although in leafhopper treatment the concentration was significantly lower than in control at 96 and 144 hours after the release, it slightly decreased after removing the leafhopper (144 and 192 hours in early treatment) and increased in control at 192 hours (Fig. 1A). The concentration of iP was not significantly different between control and treatments in the susceptible variety (Fig. 1C) but that of tZ was significantly higher in leafhopper treatment than in control at 96 and 192 hours (Fig. 1E). After removal of leafhoppers, tZ concentration slightly decreased (early treatment at 144 hours) and exposure to leafhoppers increased the tZ concentration (late treatment at 192 hours) (Fig. 1E).

**Figure 2:**
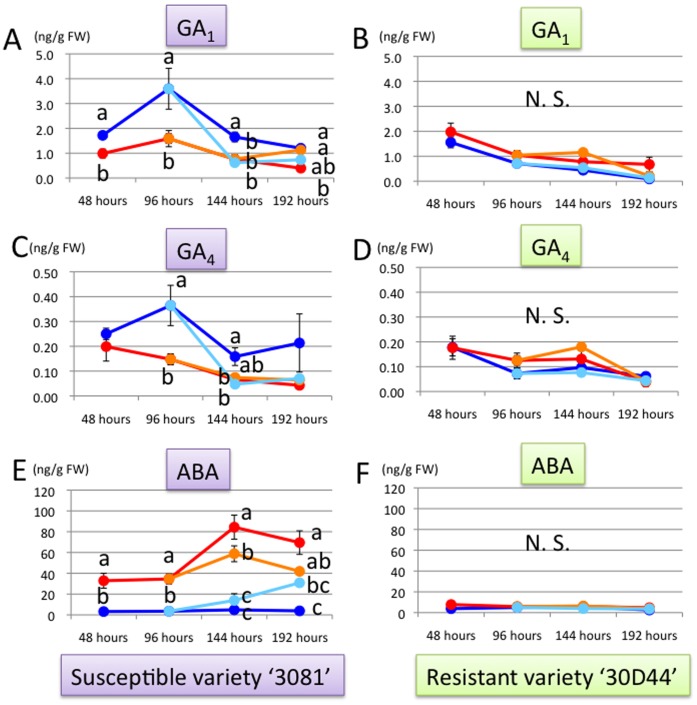
Changes in GA_1_, GA_4_ and ABA concentrations in the third leaf of maize. (A) GA_1_ concentration (ng/FW) in the variety ‘3081’. (B) GA_1_ concentration (ng/FW) in the variety ‘30D44’. (C) GA_4_ concentration (ng/FW) in the variety ‘3081’. (D) GA_4_ concentration (ng/FW) in the variety ‘30D44’. (E) ABA concentration (ng/FW) in the variety ‘3081’. (F) ABA concentration (ng/FW) in the variety ‘30D44’. Line colors indicate leafhopper treatment for eight days (red lines), control (blue lines), early leafhopper treatment for four days (orange lines), and late leafhopper treatment for four days (light blue lines). Different letters indicate significant differences between control and treatments at each time point (ANOVA; followed by Tukey’s HSD test at 144 and 192 hours).

**Figure 3:**
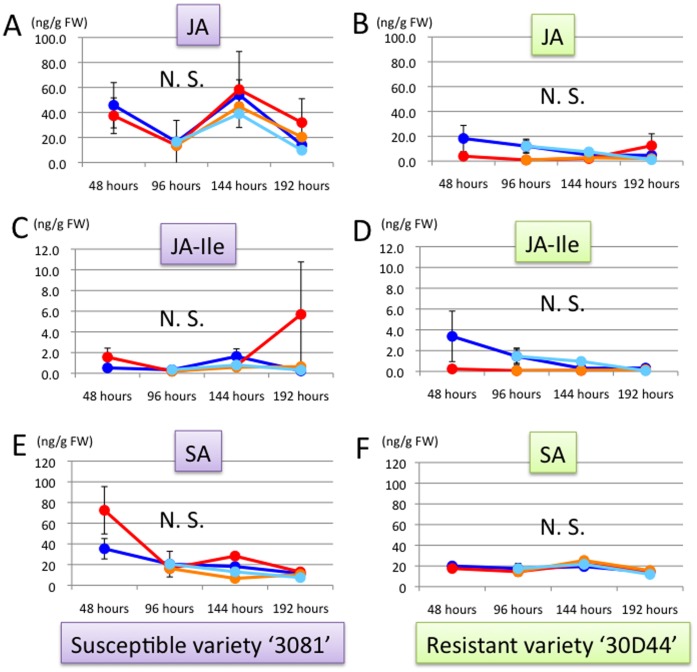
Changes in JA, JA-Ile and SA concentrations in the third leaf of maize. (A) JA concentration (ng/FW) in the variety ‘3081’. (B) JA concentration (ng/FW) in the variety ‘30D44’. (C) JA-Ile concentration (ng/FW) in the variety‘3081’. (D) JA-Ile concentration (ng/FW) in the variety ‘30D44’. (E) SA concentration (ng/FW) in the variety ‘3081’. (F) SA concentration (ng/FW) in the variety ‘30D44’. Line colors indicate leafhopper treatment for eight days (red lines), control (blue lines), early leafhopper treatment for four days (orange lines), and late leafhopper treatment for four days (light blue lines). Different letters indicate significant differences between control and treatments at each time point (ANOVA; followed by Tukey’s HSD test at 144 and 192 hours).

Concentrations of GA_1_ and GA_4_ were significantly lower in leafhopper treatment than in control (Fig. 2AC). In late treatment, GA concentrations immediately decreased after the exposure to leafhoppers (at 144 hours) (Fig. 2AC). In early treatment, GA_1_ concentration significantly increased at 192 hours ( = 96 hours after the removal of leafhoppers) (Fig. 2A). ABA concentration was most clearly affected by leafhopper treatments. In the treatment, ABA concentration was ten to 30 times higher than in control (Fig. 2E). The concentration relatively decreased after the removal of leafhoppers (at 144 and 192 hours of early treatment) and started to increase soon after exposure to leafhoppers (at 144 and 192 hours of late treatment.) (Fig. 2E). Concentrations of JA (Fig. 3A), JA-Ile (Fig. 3C), and SA (Fig. 3E) were not significantly different among control and treatments.

In the resistant variety 30D44, leafhopper treatments did not affect the phytohormone concentrations on the third leaf (Figs 1BDF, 2BDF and 3BDF), except for iP concentration at 96 hours (Fig. 1D). Particularly, increase in the ABA concentration was not detected at all in leafhopper treatments of 30D44 (Fig. 2F).

**Figure 4:**
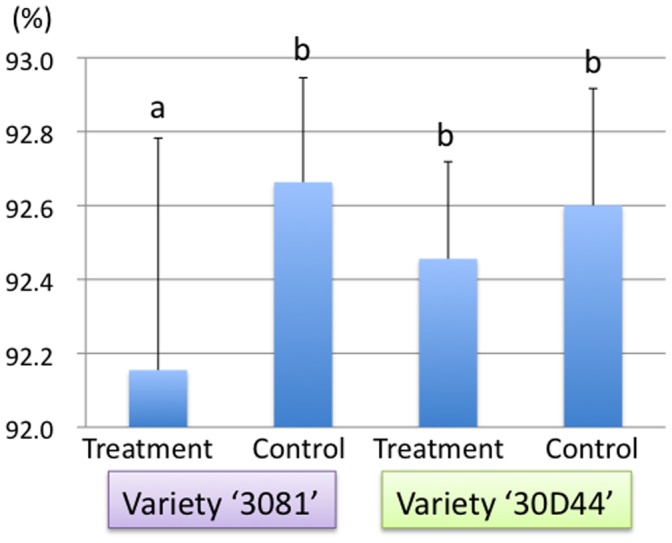
Water contents (%) of the third leaves of maize varieties ‘3081’ susceptible to *C. bipunctata* and ‘30D44’ resistant to *C. bipunctata*.

In the susceptible variety, water content at 192 hours after the release of leafhopper was significantly lower in treatment than in control, but no significant difference was detected between them in the resistant variety (Fig. 4).

## Discussion

Previous studies have reported that exogenous auxins can induce gall-like tissues on plants [36]–[38], and identified IAA in gall-inducing insects [39]–[43]. High concentrations of IAA in gall-inducing larvae of Tephritidae (Diptera) and Gelechiidae (Lepidoptera) associated with *Solidago altissima* (Asteraceae) were reported in some recent studies [44]–[45]. Moreover, sawfly larvae (Hymenoptera: Tenthredinidae) inducing leaf galls on *Salix japonica* (Salicaceae) have the ability to synthesize IAA [8]. In the present study, IAA concentration in the third leaves tended to reduce in leafhopper treatment at 96 and 144 hours, but no significant differences were detected at 48 and 192 hours. In addition, late treatment did not exhibit differences in IAA concentration (Fig. 1A). No clear differences in the IAA concentration in this study may imply that IAA is not related to the gall induction by *C. bipunctata*. However, the results do not necessarily preclude the possibility that IAA has an important role in gall induction by *C. bipunctata*. Biosynthesis of IAA in plant tissues transformed by *Agrobacterium tumefaciens* or with IAA biosynthetic genes did not elevate IAA concentrations despite the altered phenotypes in these plants [46]–[48]. No significant differences in IAA concentration may be caused by the rapid metabolism of IAA that results from feed-forward regulation of IAA-catabolizing enzymes induced by elevated IAA concentrations following either its transgenic production or endogenuous application [8]. Further studies will be needed to conclude the role of IAA in the gall induction by *C. bipunctata.*

In cytokinins, iP concentration was not significantly different in the susceptible variety but tZ concentration was significantly higher in the treatment at 96 and 192 hours (Fig. 1E). This indicates that feeding by *C. bipunctata* increases tZ concentration in extending leaves of plants fed on by the leafhopper. Selective elevation of tZ concentration may cause abnormal swelling of leaf veins in extending leaves through possible changes in auxin:cytokinin ratio, critically affecting cell division patterns and tissue differentiation of plants. In the study of gall-inducing sawfly [8], larvae contained tZ in their bodies at the concentration more than 1,000 times higher than in host willow leaves and larvae were strongly suggested to synthesize the phytohormone by themselves. Whether the increased tZ in extending maize leaves is originated from leafhoppers or endogenous to maize is worth examining in future studies.

GA_1_ and GA_4_ concentrations both significantly decreased by the feeding of leafhoppers in the susceptible variety (Figs. 2A, C). As mentioned earlier, the growth of the seedlings fed on by *C. bipunctata* are significantly stunted in the susceptible variety but seldom in the resistant variety [12]–[13], [31]–[32]. These phenomena are probably related to the growth stunting of maize induced by the leafhopper, because GAs are well known to be related to elongation growth of plants.

Notably, ABA concentration was remarkably accumulated in extending leaves of susceptible variety after feeding by the leafhopper, but this phenomenon was not observed in the resistant variety at all (Fig. 2E, F). A similar pattern of the increase of ABA in gall tissue was reported in a recent study using a gall-inducing psyllid [49], but the role of ABA in gall induction has not yet been clarified. Although ABA is known to increase following various environmental stresses such as drying stress, it is seldom known to cause morphological changes in plant tissues. In this study, we also detected significant decrease in water content only in extending leaves of the susceptible varieties after feeding by leafhoppers (Fig. 4). Increase in ABA might be a response of plants to drying stress initiated by the feeding of leafhoppers and gall induction. A study in China reported that galls induced on maize by *C. bipunctata* were abundant in relatively dried maize fields [50]. Further studies are needed to clarify the relationship between soil water condition, or humidity, and the degree of gall induction by *C. bipunctata*.

JA and SA are well-known to be related to induced resistance against herbivores and pathogens. In this study, JA and SA concentrations did not change significantly by leafhopper treatments both in susceptible and resistant varieties. A previous study revealed that the nymphal performance of *C. bipunctata* was higher in the susceptible variety of maize than the resistant variety [31]. Based on our analysis (Fig. 3), induced resistance via JA or SA pathways seem not to be activated both in susceptible and resistant varieties after feeding by the leafhopper. Therefore, the induced resistance involved in these phytohormones seems not to be related to the lower performance of *C. bipunctata* on the resistant variety.

Our study clearly indicated that tZ and ABA concentrations increase but GA_1_ and GA_4_ concentration decrease in leaves to be galled by *C. bipunctata.* Further studies analyzing the up- or down-regulation of phytohormone responsive genes in the leaves will elucidate manipulation mechanism of plant tissue by the gall-inducing leafhopper.
